# Neurogenic effects of rotarod walking exercise in subventricular zone, subgranular zone, and substantia nigra in MPTP-induced Parkinson’s disease mice

**DOI:** 10.1038/s41598-022-14823-5

**Published:** 2022-06-22

**Authors:** Yea-Hyun Leem, Jin-Sun Park, Jung-Eun Park, Do-Yeon Kim, Hee-Sun Kim

**Affiliations:** 1 Department of Molecular Medicine and Inflammation-Cancer Microenvironment Research Center, School of Medicine, Ewha Womans University, 808-1 Magok-dong, Gangseo-gu, Seoul, 07804 South Korea; 2 Department of Brain and Cognitive Sciences, Ewha Womans University, Seoul, South Korea

**Keywords:** Neuroscience, Diseases, Health care

## Abstract

Parkinson’s disease (PD) is the second most common neurodegenerative disease after Alzheimer’s disease, and its incidence is predicted to increase worldwide. Striatal dopamine depletion caused by substantia nigra (SN) degeneration is a pathological hallmark of PD and is strongly associated with cardinal motor and non-motor symptoms. Previous studies have reported that exercise increases neuroplasticity and promotes neurorestoration by increasing neurotrophic factors and synaptic strength and stimulating neurogenesis in PD. In the present study, we found that rotarod walking exercise, a modality of motor skill learning training, improved locomotor disturbances and reduced nigrostriatal degeneration in the subacute 1-methyl-4-phenyl-1,2,3,6-tetrahydropyridine (MPTP) mouse model of PD. In addition, our exercise regimen improved MPTP-induced perturbation of adult neurogenesis in some areas of the brain, including the subventricular zone, subgranular zone, SN, and striatum. Moreover, rotarod walking activated the phosphorylation of adenosine monophosphate-activated protein kinase (AMPK) and induced brain-derived neurotrophic factor (BDNF) expression in these regions. The results suggest that motor skill learning training using rotarod walking improves adult neurogenesis and restores motor performance by modulating the AMPK/BDNF pathway. Therefore, our findings provide evidence for neuroprotective effects and improved neuroplasticity in PD through motor skill learning training.

## Introduction

Parkinson’s disease (PD) is a movement disorder linked to nigrostriatal neurodegeneration and is characterized by muscular rigidity, resting tremor, bradykinesia, and postural instability. In addition to the prototypical motor traits of PD, non-motor symptoms manifest at the premotor stage of the disease, including olfactory dysfunction, sleep disturbance, mild cognitive impairment, psychiatric illness, and gastrointestinal disturbance^1–4^. These non-motor symptoms are associated with neurological abnormalities of circuits other than the nigrostriatal dopaminergic pathway^5,6^.

Adult neurogenesis is a multistage process in which newly generated neurons are integrated into pre-exiting neuronal circuitries. Adult neurogenesis is predominantly restricted to the two main neurogenic niches, the subventricular zone (SVZ) and subgranular zone (SGZ)^7,8^. This process is a crucial component of neural plasticity required for physiological brain function, and its disruption is implicated in neurodegenerative diseases, such as PD and Alzheimer’s disease (AD)^9–12^. In particular, perturbation of olfactory- and hippocampal-derived adult neurogenesis is closely linked to hyposmia, mood, and cognitive changes in PD progression^4,6,13^. In addition to neurogenic niches, some studies have revealed the presence of newly generated neurons in the substantia nigra (SN) region of the brain^14–18^. Although the findings of adult neurogenesis in the SN region are still under debate due to certain issues, such as experimental reproducibility^19^ and the differentiating tendency of progenitor cells into astrocytes rather than into neurons^16^, the aforementioned studies suggest the possibility of neuronal replacement and functional integration of new-born neuronal cells into nigrostriatal pathway against loss of dopaminergic neurons. Therefore, modulating neuronal replenishment in PD progression may be a potential therapeutic option; however, the biological mechanisms in the early stage of PD progression are poorly understood.

Exercise promotes neuroplasticity and elicits neuroprotection under physiological and pathological conditions. Multiple lines of evidence have demonstrated the beneficial effects of exercise on PD pathophysiology including the following: (1) improvement of striatal dopamine (DA) release and transmission^20,21^, (2) reversion of aberrant glutamatergic synaptic drive in the corticostriatal pathway^22^, (3) elevated expression of neurotrophic factors such as brain-derived neurotrophic factor (BDNF)^23,24^, and (4) improvement in mitochondrial dysfunction such as biogenesis- and fusion-associated abnormalities^25,26^. Regarding adult neurogenesis, several studies have shown that endurance exercise improves MPTP-induced deficits in adult hippocampal neurogenesis^27,28^. However, most of the above-mentioned animal studies adopted unskilled aerobic exercise, such as treadmill running or wheel running, rather than skilled exercise for physical intervention in PD.

In clinical practice, physical rehabilitation for patients with PD primarily targets postural stabilization and mobility-related functional gains, which are helpful for improving balance and gait function and reducing the fall rate. Unlike aerobic exercise, skilled exercise aims to achieve a specific dexterity, which induces proper temporal and spatial performance. Despite the disease-modifying role of skilled exercise in PD, the neurogenic impact of this type of exercise on PD pathophysiology has not been well studied. Therefore, in the present study, we investigated the potential role of rotarod walking exercise, a form of motor skill learning training, in PD-linked motor symptoms and in adult neurogenesis in the SVZ, SGZ, SN, and striatum regions of MPTP-induced PD mice.

## Materials and methods

### Reagents and antibodies

MPTP was purchased from Tokyo Chemical Industry Co., Ltd. (Tokyo, Japan). 5-Bromo-2-deoxyuridine (BrdU) was obtained from Sigma-Aldrich (St. Louis, MO, USA). Sodium pentobarbital was purchased from Hanlim Pharm Co., Ltd. (Seoul, South Korea). Biotinylated secondary antibodies (1:200, Cat# BA-1000) and fluorochrome-conjugated secondary antibodies (1:500, Cat# DI-3094, DI-1094, DI-2594), diaminobenzidine tetrahydrochloride, and antifade reagent were obtained from Vector Laboratories (Burlingame, CA, USA). The following primary antibodies were used in this study: anti-tyrosine hydroxylase (1:2000, Cat# 58844), anti-Ki67 (1:500, Cat# 9129), anti-doublecortin (1: 500, Cat# 4604) and anti-p-AMPK (1:1000, Cat# 2535) antibodies from Cell Signaling Technology, Inc. (Danvers, MA, USA) and anti-BDNF (1:1000, Cat# ab203573) and anti-BrdU (1:250, Cat# ab6326) from Abcam (Cambridge, UK).

### Animals

Adult male C57BL/6 mice (7 weeks of age) were purchased from Orient Bio Inc. (Seongnam, South Korea), a branch of Charles River Laboratories. Mice were maintained at 21 °C under a 12-h light:12-h dark cycle and had ad libitum access to water and rodent chow. Every effort was made to minimize animal suffering. All experiments were performed in accordance with the National Institutes of Health (NIH) and Ewha Womans University guidelines for laboratory animal care and use, and the study was approved by the Institutional Animal Care and Use Committee of the Medical School of Ewha Womans University (#EUM 20-022). The study was carried out in compliance with the ARRIVE guidelines.

### Experimental procedure

All male mice were subjected to pre-training for 3 days (rotarod and pole tests), and subsequently injected with BrdU. Following BrdU injection, MPTP and probenecid were administrated for 5 consecutive days. Exercised mice were forced to undergo rotarod walking training for 4 weeks, and behavioral tests were assessed 2 days after the completion of exercise regimen (the rotarod test was performed on the test day 1 and the pole test was implemented, followed by PaGE test 2 h later on the test day 2).

### MPTP administration

Mice were randomly divided into four groups: CON, control; M/P, MPTP + probenecid; M/P + Ex, M/P + rotarod exercise; and Ex, rotarod exercise (CON, N = 10; M/P, N = 12; M/P + Ex, N = 13; Ex, N = 10). Mice were intraperitoneally administered MPTP (25 mg/kg/injection; on day 1–2, twice injections/day, 6 h-interval; on day 3–5, one injection/day) 30 min after probenecid (250 mg/kg/day) injection for 5 days^29^. One day after MPTP exposure, the mice were forced to perform a rotarod walking exercise for 28 days. Mice were subjected to behavioral tests 2 and 3 days after the last exercise treatment. For histological analysis, mice were decapitated 2 days after completion of the behavioral tests.

### BrdU administration

BrdU is a synthetic analog similar to thymidine that is incorporated during the S-phase of the cell cycle, and is commonly used as a marker for cell proliferation in living tissue. BrdU stock solution was prepared in 0.02 N NaOH to completely dissolve BrdU powder (10 mg/mL) and stored at − 20 °C. At the time of administration, we used 1:3 BrdU: 0.9% normal saline. Mice were injected with BrdU intraperitoneally (50 mg/kg, i.p.) for 5 consecutive days a day before MPTP treatment (slightly modified from a previous study^30^).

### Rotarod walking exercise protocol

The rotarod walking exercise was delivered at a set time from 1700 to 2000 for four weeks (5 days/week). Mice were kept on the resting rod of a rotarod machine for at least 3 min. The speed of the rotarod was gradually increased on a weekly basis throughout the exercise period (8–20 rpm, 20–38 min/day, 4 weeks). All exercised mice were pre-exercised at 8 rpm for 5 min prior to the principal exercise administration and were cooled down at 8 rpm for 3 min after the principal exercise completion throughout the exercise period. On days 1–3 in the first week, the rod speed was set at 10 rpm for 30 min. On day 4–5 in the first week, the speed was set at 10–12 rpm for 30 min. In the second week, the mice were run at 11–15 rpm for 30 min. In the third week, the rod speed was set at 13–18 rpm for 30 min. In the fourth week, the mice were run at 16–20 rpm for 30 min. The mice who were not subjected to exercise stayed on a resting rod for 3 min.

### Behavioral tests

To assess the motor coordination of the mice, an accelerated rotarod test was performed. Prior to the MPTP treatment, the mice were trained for four consecutive days, with three trials per day, each day with an intersession interval of 15 min. On test day, mice were placed on the resting drum (3-cm diameter) of a rotarod apparatus (Harvard Apparatus, MA, USA) for at least 1 min. The speed of the rotarod was accelerated from 4 to 40 rpm over a 300-s period. The mice were subjected to three trials with 15 min intervals between trials. The retention time of the rod in each trial was recorded. To evaluate akinesia, a pole test (50 cm in height, 0.5 cm in diameter, 120 s) was implemented. Initially, all mice were trained to successfully descend from the top to the bottom of the pole. The time taken for each mouse to descend the pole was recorded. Each mouse was subjected to three trials, and the average was recorded. The paw grip endurance test (PaGE) was used to assess the muscular endurance of the paw. Thirty percent of the body weight was attached to the base of the tail with form tape. Each mouse was placed on the wire lid (15 cm × 15 cm), and the lid was gently turned upside down to elicit gripping with four paws. The time spent on the inverted wire lid was also recorded. Each mouse was subjected to three trials, and the average was recorded.

### Histological analysis

Mice were anesthetized with sodium pentobarbital (80 mg/kg) to induce rapid and prolonged anesthesia. Mice were then perfused transcardially, their brains were removed, and 40-µm-thick sections were prepared using a cryotome. We analyzed five sections for each region of the SVZ, striatum, and DG (every six sections/brain) and four sections for the SN (every three sections/brain). For immunohistochemistry (IHC) analysis, the sections were subjected to endogenous peroxidation inactivation with 3% hydrogen peroxide (H_2_O_2_), and non-specific binding was blocked with 4% bovine serum albumin (BSA). Initially, the sections were incubated overnight with primary antibodies and then incubated with biotinylated secondary antibodies for 1 h at 25 °C on the following day. The sections were subsequently incubated with avidin–biotin–HRP complex reagent solution for 1.5 h, and a peroxidase reaction was performed using diaminobenzidinetetrahydrochloride. For double immunofluorescence (IF) analysis, non-specific binding was blocked, and the sections were incubated with primary antibodies, followed by fluorochrome-conjugated secondary antibodies. For BrdU double IF analysis, the sections were incubated with 2 M HCl for 30 min at 37 °C to denature the DNA, followed by incubation with 0.1 M borate buffer (pH 9.0) for 10 min at 24 °C to neutralize the DNA. Non-specific binding was blocked, and the sections were incubated with primary antibodies, followed by fluorochrome-conjugated secondary antibodies. The tissue was then mounted using an anti-fade reagent. Digital images of IHC and IF staining were captured using a Leica DM750 microscope (Leica Microsystems, Nussloch, Germany). Quantification was performed using ImageJ (version 1.8.0, NIH Image Engineering, Bethesda, MD, USA) and AxioVision (version 4.8.2. SP2, Carl Zeiss Microscopy GmbH, Jena, Germany). Each immunoreactive measure was analyzed from the striatum and SVZ (the region from 1.53 to 0.01 mm from the bregma), dorsal HP (− 1.31 to − 2.53 mm), ventral HP (− 2.91 to − 3.51 mm), and SN areas (− 3.07 to − 3.63 mm). For cell counting, high magnitude images (200×) were obtained from each slide, and cells were counted according to ImageJ’s instruction, using ImageJ’s plugins, Colour Deconvolution and Cell Counter. In brief, an image was selected from Colour Deconvolution. In ImageJ’s tools, the threshold of the image was adjusted using the Threshold tool and then a binary image was created using the Binary tool. Thereafter, using the Watershed tool, imaged separation lines among cells were generated by pixel-based segmentation. Finally, cells were counted using the Analyze Particles tool. To reconfirm, cells were re-counted using the Cell Counter, ImageJ’s plugin.

### Statistical analysis

Statistical analyses were performed using SPSS for Windows (version 18.0; SPSS Inc., Chicago, IL, USA). Differences among the groups were analyzed using one-way analysis of variance (ANOVA). Post-hoc comparisons were conducted using the least significant difference (LSD) test. All values are presented as the mean ± standard error of the mean (SEM). Differences were considered statistically significant at p < 0.05.

## Results

### Rotarod exercise improved MPTP-induced impaired movement performance and defect of nigrostriatal dopaminergic circuitry

**Figure 1:**
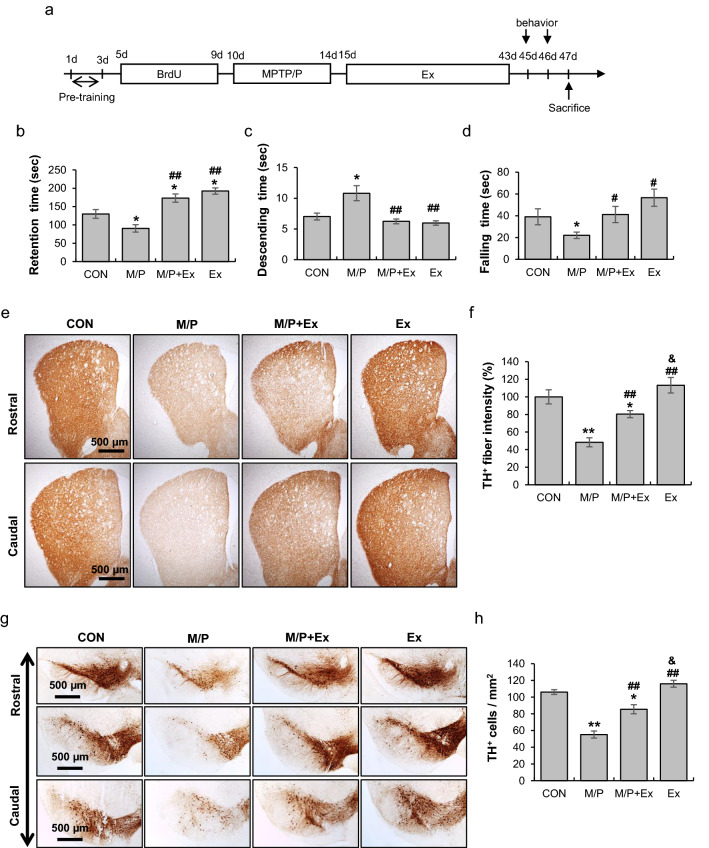
Rotarod walking exercise improved impaired motor performance and defects of nigrostriatal dopaminergic pathway in M/P-treated mice. (**a**) The experimental procedure: mice were treated with BrdU for 5 days and the next 5 days with MPTP/probenecid (M/P), and were subsequently subjected to rotarod exercise for 4 weeks. Behavioral tests were then evaluated 2 days after exercise completion. Mice were sacrificed 1 day after behavioral tests. (**b**) 4-week of exercise extended M/P-induced decrease in retention time on the accelerated rotarod test (N = 10 in each group) (**c**) 4-week of exercise delayed M/P-induced increase in descending time on the pole test (N = 10 in each group) (**d**) 4-week of exercise delayed M/P-induced decrease in falling time on the PaGE test (N = 10 in each group) (**e**, **f**) Photomicrographs (**e**) and quantification (**f**) of the immunoreactivity of striatal TH^+^ particles (N = 8 in each group, 40 × of magnification) showing that exercise improved the M/P-induced decrease in TH^+^ projections in the striatum. (**g**, **h**) Photomicrographs (**g**) and quantification (**h**) of the immunoreactivity of SN TH^+^ cells (N = 8 in each group, 40 × of magnification) showing that exercise improved the M/P-induced decrease in TH^+^ cells in the SN. Data are presented as mean ± SEM. *p < 0.05, vs. CON; **p < 0.01, vs. CON; ^#^p < 0.05, vs. M/P; ^##^p < 0.01, vs. M/P; ^&^p < 0.05, vs M/P + Ex. CON, control; M/P, MPTP/probenecid; M/P + Ex, M/P + exercise; Ex, exercise, TH, tyrosine hydroxylase.

Mice were initially treated with BrdU for 5 days and with MPTP + probenecid (M/P) for the next 5 days. Subsequently, they were subjected to rotarod exercise for 4 weeks (Fig. 1a). M/P treatment significantly reduced retention time in the rotarod; meanwhile, exercise substantially enhanced the retention time in M/P-treated mice. The retention time of exercised mice was higher than that of the control mice (each group N = 10, F_3__,__36_ = 19.44, p < 0.01, Fig. 1b). The descending time of M/P group was extended relative to that of control group, while the time was shortened by exercise regimen in the pole test (each group N = 10, F_3__,__36_ = 9.62, p < 0.01, Fig. 1c). Moreover, the time that the mouse hung onto the lid in the PaGE test was significantly reduced with M/P treatment, which was improved by exercise (each group N = 10, F_3__,__36_ = 4.46, p < 0.01, Fig. 1d). The results suggest that long-term motor skill learning training, such as rotarod walking exercise, can improve reduced muscular endurance capacity, as well as impaired motor coordination and dyskinesia, in the subacute MPTP model for PD.

IHC analysis showed that the exercise regimen improved M/P-induced decrease in TH^+^ immunoreactivity in the striatum region; however, the immunoreactivity of the M/P + Ex group did not amount to that of CON and Ex groups (each group N = 8, F_3,28_ = 17.19, p < 0.01, Fig. 1e,f). Moreover, the number of SNpc TH^+^ cells in exercised mice increased in M/P-treated mice, despite the fact that the TH^+^ cells of M/P + Ex mice were lower than those of the CON and Ex groups (each group N = 8, F_3,28_ = 47.95, p < 0.01, Fig. 1g,h). The results indicate that long-term motor skill learning training prevents further insults or has a beneficial impact on the nigrostriatal dopaminergic circuitry in MPTP-induced toxicity.

### Rotarod exercise improved MPTP-induced deficits in neuronal differentiation in the SVZ area

**Figure 2:**
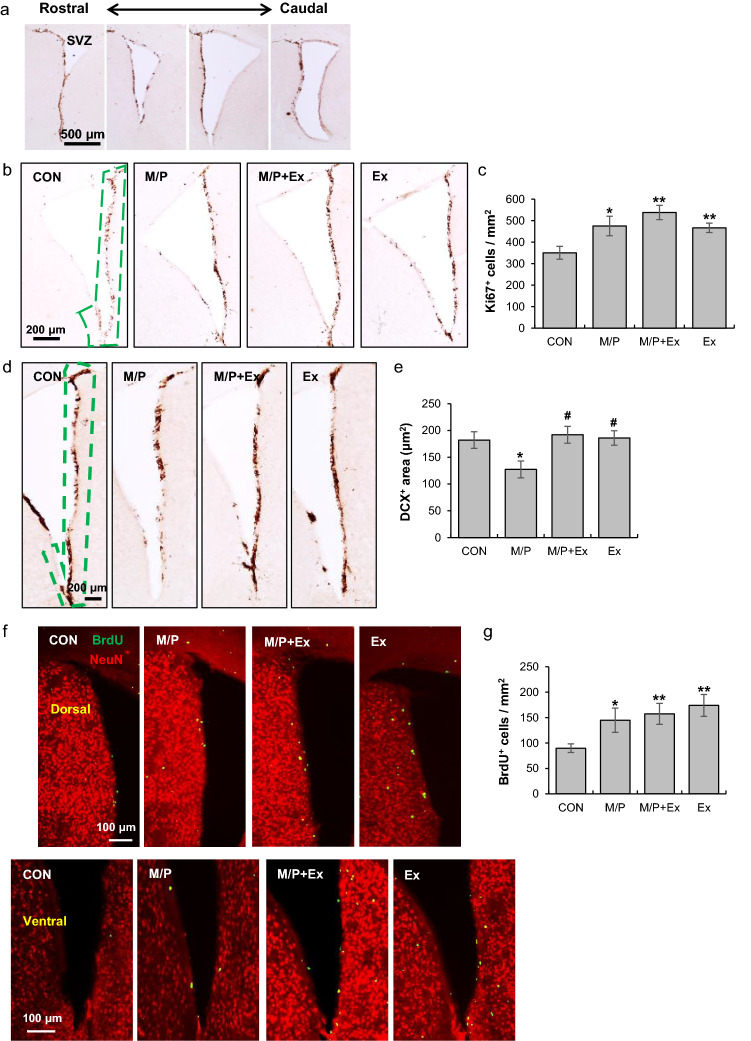
Rotarod walking exercise reduced the deficits of neuronal differentiation in the SVZ region of M/P-treated mice. (**a**) Photomicrographs of the SVZ areas analyzed by histological analysis (40 × of magnification). (**b**) Photomicrographs of the immunoreactivity of SVZ Ki67^+^ cells (CON, N = 6; M/P, N = 7; M/P + Ex, N = 7; Ex, N = 6, 40 × of magnification). (**c**) Quantification of the immunoreactivity of SVZ Ki67^+^ cells showing that M/P and exercise increased the number of Ki67^+^ cells. (**d**) Photomicrographs of the immunoreactivity of SVZ DCX^+^ cells (CON, N = 6; M/P, N = 7; M/P + Ex, N = 7; Ex, N = 6, 40 × of magnification). (**e**) Quantification of the immunoreactivity of SVZ DCX^+^ cells showing that exercise restored the M/P-induced decrease in DCX^+^ cells. (**f**) Photomicrographs of the double fluorescent staining of SVZ BrdU^+^ and NeuN^+^ cells (CON, N = 8; M/P, N = 9; M/P + Ex, N = 9; Ex, N = 8, 100 × of magnification). (**g**) Quantification of the fluorescent staining of SVZ BrdU^+^ cells showing that M/P and exercise increased the number of BrdU^+^ cells. Data are presented as mean ± SEM. *p < 0.05, vs. CON; **p < 0.01, vs. CON; ^#^p < 0.05, vs. M/P. DCX, doublecortin.

Based on the finding that the rotarod exercise improved behavioral and cellular defects in MPTP-induced neurotoxicity, we explored the neurogenic effects of long-term rotarod exercise on adult neurogenesis in several structures, including the SVZ, SGZ, and SN regions. In the SVZ, the proliferating ability (measuring an endogenous proliferating marker, Ki67) of the three groups (M/P, M/P + Ex, Ex) was significantly enhanced compared to that of the control (CON, N = 6; M/P, N = 7; M/P + Ex, N = 7; Ex, N = 6, F_3,23_ = 5.29, p < 0.01, Fig. 2a–c). The rotarod exercise restored the M/P-induced decrease in doublecortin (DCX), a neuroblast marker (CON, N = 6; M/P, N = 7; M/P + Ex, N = 7; Ex, N = 6, F_3,28_ = 3.93, p < 0.05, Fig. 2d,e). Moreover, to assess the survival of neural precursor cells, we detected BrdU-pre-labeled cells after M/P treatment and exercise regimen completion. Similar to Ki67 data, BrdU^+^ cells in the M/P, M/P + Ex, and Ex groups were higher than those in the control group (CON, N = 8; M/P, N = 9; M/P + Ex, N = 9; Ex, N = 8, F_3,31_ = 3.42, p < 0.05, Fig. 2f,g). These findings indicate that M/P treatment enhances cell proliferation and survival; meanwhile, neuronal differentiation is attenuated. However, long-term motor skill learning training enhances neuronal differentiation, as well as the proliferation and survival of neural precursor cells.

### Rotarod exercise induced AMPK phosphorylation and BDNF expression in SVZ area

**Figure 3:**
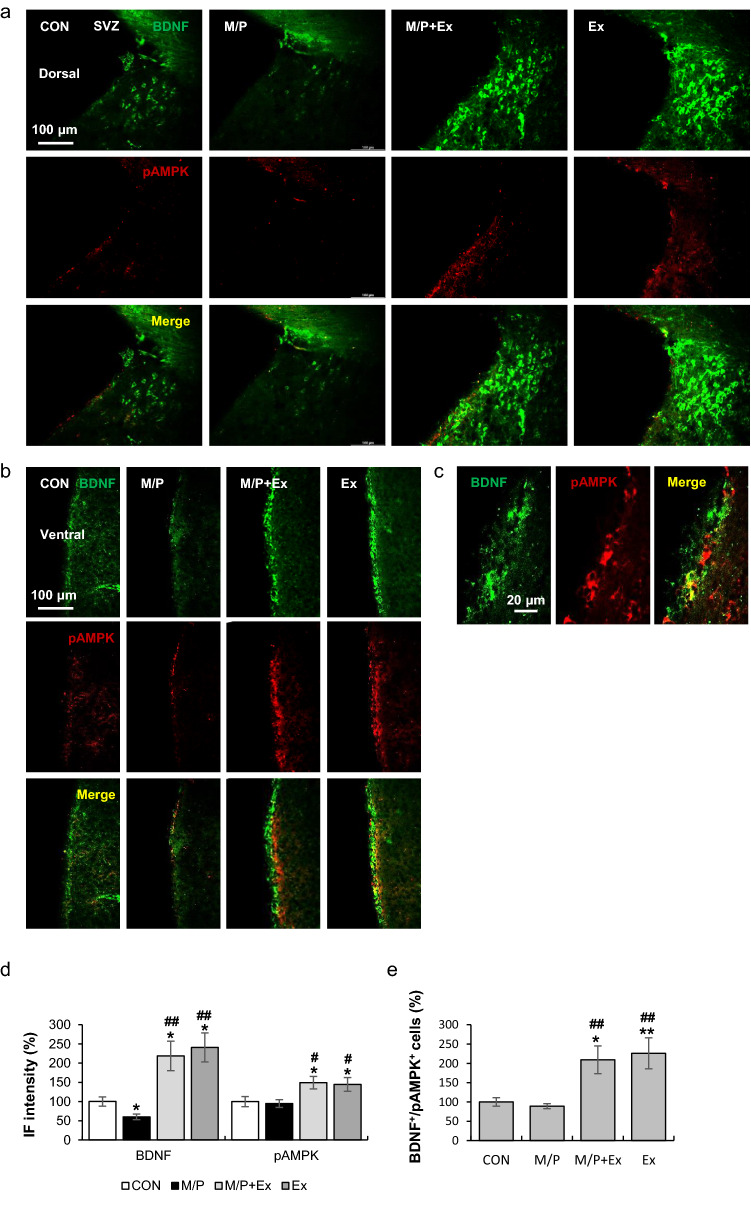
Rotarod walking exercise activated the AMPK/BDNF pathway in the SVZ area of M/P-treated mice. (**a**) Photomicrographs of the double fluorescent staining of dorsal SVZ BDNF^+^ and pAMPK^+^ cells (CON, N = 8; M/P, N = 9; M/P + Ex, N = 9; Ex, N = 8, 200 × of magnification). (**b**) Photomicrographs of the double fluorescent staining of ventral SVZ BDNF^+^ and pAMPK^+^ cells (CON, N = 8; M/P, N = 9; M/P + Ex, N = 8; Ex, N = 9, 200 × of magnification). (**c**) Representative photomicrograph of the high magnitude of SVZ BDNF^+^/pAMPK^+^ cells (400 × of magnification). (**d**) Quantification of the fluorescent intensity of SVZ BDNF^+^ and pAMPK^+^ particles showing that exercise restored the M/P-induced decrease in BDNF^+^, and enhanced the pAMPK^+^ IF intensity, regardless of MPTP treatment (CON, N = 8; M/P, N = 9; M/P + Ex, N = 9; Ex, N = 8). (**e**) Quantification of the fluorescent staining of SVZ BDNF^+^/pAMPK^+^ cells showing that exercise increased the number of BDNF^+^/pAMPK^+^ cells, regardless of MPTP treatment. Data are presented as mean ± SEM. *p < 0.05, vs. CON; **p < 0.01, vs. CON; ^#^p < 0.05, vs. M/P; ^##^p < 0.01, vs. M/P.

Multiple lines of evidence have demonstrated the beneficial effects of exercise via AMPK-mediated BDNF induction in the brain. Therefore, double staining for pAMPK and BDNF was conducted in several regions, including the SVZ, SGZ, and SN regions. We found that exercise substantially improved the M/P-induced reduction of BDNF^+^ immunoreactivity in the SVZ and its adjacent area, in which BDNF protein levels in M/P + Ex mice were similar to those of Ex (CON, N = 8; M/P, N = 9; M/P + Ex, N = 9; Ex, N = 8, F_3,28_ = 10.13, p < 0.01, Fig. 3a–d). The pAMPK^+^ immunoreactivity in the M/P + Ex and Ex groups was higher than that in the CON and M/P groups; however, no significant difference was found between CON and M/P mice (CON, N = 8; M/P, N = 9; M/P + Ex, N = 9; Ex, N = 8, F_3,28_ = 4.10, p < 0.05, Fig. 3a–d). Furthermore, BDNF^+^/pAMPK^+^ cells of exercised mice were markedly increased regardless of M/P treatment (CON, N = 8; M/P, N = 9; M/P + Ex, N = 9; Ex, N = 8, F_3,28_ = 10.13, p < 0.01, Fig. 3e). These results suggest that long-term motor skill learning training facilitates AMPK-mediated BDNF induction, which may contribute to adult neurogenesis in the SVZ area.

### Rotarod exercise improved MPTP-induced deficits of neuronal differentiation of the ventral SGZ area

**Figure 4:**
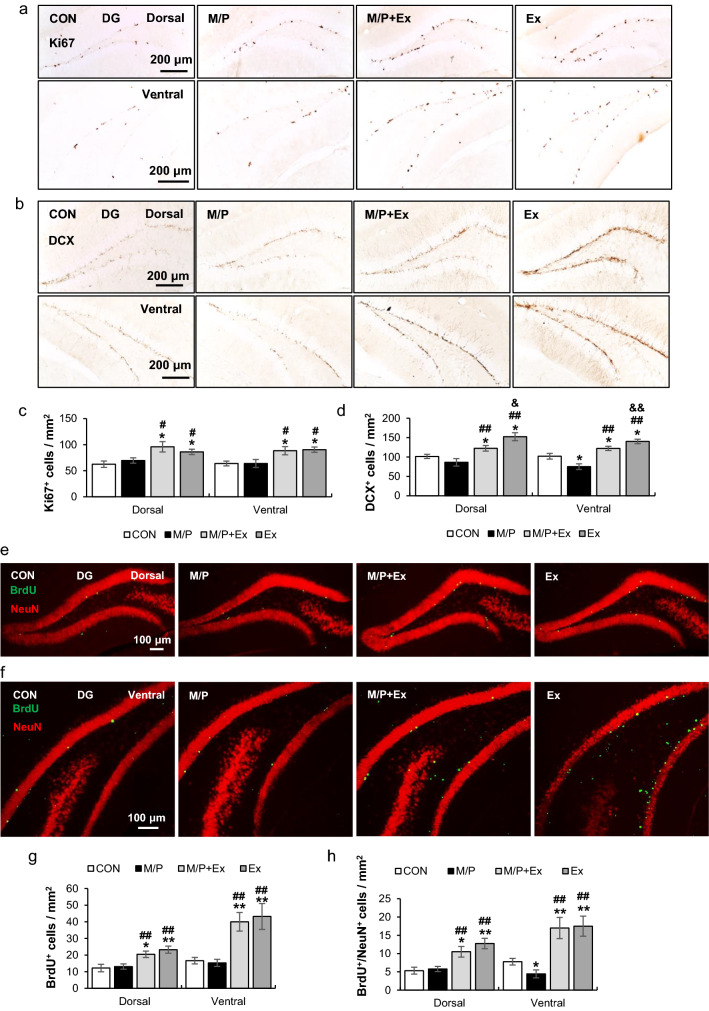
Rotarod walking exercise reduced the decline of neuronal differentiation in the ventral SGZ region of M/P-treated mice. (**a**) Photomicrographs of the immunoreactivity of SGZ Ki67^+^ cells (CON, N = 6; M/P, N = 7; M/P + Ex, N = 7; Ex, N = 6, 100 × of magnification). (**b**) Photomicrographs of the immunoreactivity of SGZ DCX^+^ cells (CON, N = 6; M/P, N = 7; M/P + Ex, N = 7; Ex, N = 6, 100 × of magnification). (**c**) Quantification of the immunoreactivity of SGZ Ki67^+^ cells showing that exercise increased the number of Ki67^+^ cells, regardless of MPTP treatment. (**d**) Quantification of the immunoreactivity of SGZ DCX^+^ cells showing that exercise restored the M/P-induced decrease in DCX^+^ cells in the ventral SGZ, and increased the number of DCX^+^ cells in the dorsal region, regardless of MPTP treatment. (**e**) Photomicrographs of the double fluorescent staining of dorsal SGZ BrdU^+^ and NeuN^+^ cells (CON, N = 8; M/P, N = 9; M/P + Ex, N = 9; Ex, N = 8, 100 × of magnification). (**f**) Photomicrographs of the double fluorescent staining of ventral SGZ BrdU^+^ and NeuN^+^ cells (CON, N = 8; M/P, N = 9; M/P + Ex, N = 9; Ex, N = 8, 100 × of magnification). (**g**) Quantification of the fluorescent staining of SGZ BrdU^+^ cells showing that exercise increased the number of BrdU^+^ cells, regardless of MPTP treatment. (**h**) Quantification of the fluorescent staining of SGZ BrdU^+^/NeuN^+^ cells showing that exercise restored the M/P-induced decrease in BrdU^+^/NeuN^+^ cells in the ventral SGZ, while increasing the number of BrdU^+^/NeuN^+^ cells in the dorsal region, regardless of MPTP treatment. Data are presented as mean ± SEM. *p < 0.05, vs. CON; **p < 0.01, vs. CON; ^#^p < 0.05, vs. M/P; ^##^p < 0.01, vs. M/P; ^&^p < 0.05, vs. M/P + Ex; ^&&^p < 0.01, vs. M/P + Ex.

In determining the proliferation of neural precursor cells using Ki67^+^ cells in the SGZ region, exercise substantially enhanced Ki67^+^ cells, regardless of the M/P treatment, in the dorsal and ventral SGZ regions, in which Ki67^+^ cells of the CON group were similar to those of M/P in both regions (CON, N = 6; M/P, N = 7; M/P + Ex, N = 7; Ex, N = 6: for dorsal F_3,21_ = 4.88, p < 0.05; for ventral F_3,21_ = 5.46, p < 0.01; Fig. 4a,c). In assessing neuronal differentiation, the number of DCX^+^ cells in the M/P + Ex and Ex groups significantly increased compared to those of CON and M/P groups, and the cells in the dorsal region of CON mice were comparable to those of M/P mice (each group N = 8: for dorsal F_3,28_ = 13.42, p < 0.01, Fig. 4b,d). Unlike in the dorsal region, M/P treatment decreased DCX^+^ cells in the ventral region, which were recovered by exercise. In addition, the DCX^+^ cells of the Ex group were higher than those of the CON group (each group N = 8: for dorsal F_3,28_ = 15.58, p < 0.01, Fig. 4b,d). These results suggest that M/P caused region-specific damage, especially in the ventral SGZ. Long-term motor skill training promotes adult neurogenesis under M/P-induced neurotoxicity in both dorsal and ventral SGZ regions.

To revalidate the neurogenic effects of rotarod exercise on the SGZ area in M/P neurotoxicity, co-localized BrdU^+^ /NeuN^+^ cells were evaluated. Exercise enhanced BrdU^+^ cells in the dorsal and ventral SGZ regions compared to those in CON mice regardless of the M/P treatment, in which there was no difference in BrdU^+^ cells between CON and M/P mice (CON, N = 8; M/P, N = 9; M/P + Ex, N = 9; Ex, N = 8: for dorsal F_3,30_ = 7.38, p < 0.01; for ventral F_3,30_ = 10.12, p < 0.01; Fig. 4e–g). Moreover, exercise augmented BrdU^+^ overlaid with NeuN^+^ cells compared to those in the CON group regardless of the M/P treatment in the dorsal SGZ region, in which the cells of the CON group were comparable to those of the M/P group (CON, N = 8; M/P, N = 9; M/P + Ex, N = 9; Ex, N = 8, F_3,30_ = 9.73, p < 0.01, Fig. 4e,h). In the ventral SGZ region, M/P treatment caused a decrease in BrdU^+^/NeuN^+^ cells, and exercise inversely enhanced these cells regardless of the M/P treatment (CON, N = 8; M/P, N = 9; M/P + Ex, N = 9; Ex, N = 8, F_3,30_ = 10.59, p < 0.01, Fig. 4f,h). This result suggests that the ventral SGZ regions may be vulnerable to M/P toxicity, and long-term skill learning training facilitates the survival and neuronal differentiation of neural precursor cells in the SGZ area.

### Rotarod exercise induced AMPK phosphorylation and BDNF expression in SGZ area

**Figure 5:**
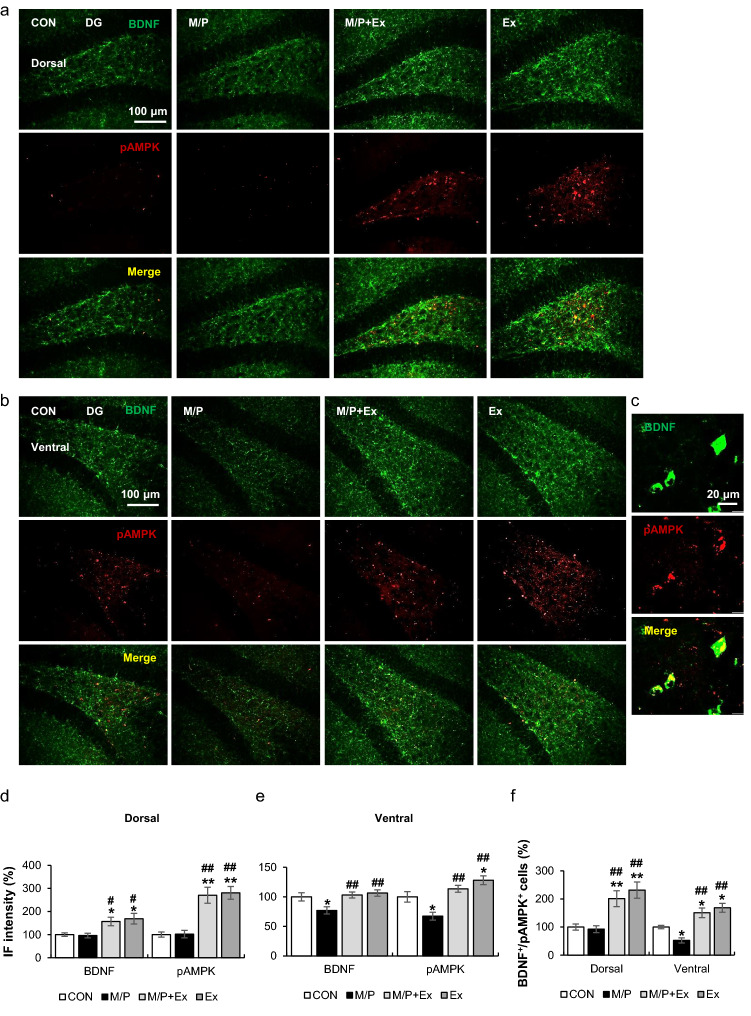
Rotarod walking exercise activated the AMPK/BDNF pathway in the SGZ area of M/P-treated mice. (**a**) Photomicrographs of the double fluorescent staining of dorsal SGZ BDNF^+^ and pAMPK^+^ cells (N = 8 in each group, 200 × of magnification). (**b**) Photomicrographs of the double fluorescent staining of ventral SGZ BDNF^+^ and pAMPK^+^ cells (N = 8 in each group, 40 × of magnification). (**c**) Representative photomicrograph of the high magnitude of SGZ BDNF^+^ and pAMPK^+^ cells (400 × of magnification). (**d**, **e**) Quantification of the fluorescent intensity of SGZ BDNF^+^ and pAMPK^+^ particles showing that exercise restored the M/P-induced decrease in BDNF^+^ and pAMPK^+^ cells in the ventral SGZ, and increased the number of BDNF^+^ and pAMPK^+^ cells in the dorsal region, regardless of MPTP treatment. (**f**) Quantification of the fluorescent staining of SGZ BDNF^+^/pAMPK^+^ cells, showing that exercise restored the M/P-induced decrease in BDNF^+^/pAMPK^+^ cells in the ventral SGZ, and increased the number of BDNF^+^/pAMPK cells in the dorsal region, regardless of MPTP treatment. Data are presented as mean ± SEM. *p < 0.05, vs. CON; **p < 0.01, vs. CON; ^#^p < 0.05, vs. M/P; ^##^p < 0.01, vs. M/P.

Similar to the SVZ area, exercise increased the immunoreactivity of BDNF^+^ and pAMPK^+^ particles relative to those in the CON and M/P groups in the dorsal SGZ region (each group N = 8; for BDNF F_3,28_ = 5.51, p < 0.01; for pAMPK F_3,28_ = 17.33, p < 0.01; Fig. 5a,d). Unlike the dorsal part, in the ventral SGZ region, M/P treatment reduced the immunoreactivity of BDNF^+^ and pAMPK^+^ particles, which was reversed by exercise (each group N = 8; for BDNF F_3,28_ = 5.00, p < 0.01; for pAMPK F_3,28_ = 12.80, p < 0.01; Fig. 5b,e). Furthermore, exercise increased the colocalized BDNF^+^/pAMPK^+^ cells compared to those of CON and M/P mice in the dorsal and ventral parts of the SGZ area, in which the colocalized cells of the M/P group were lower than those of the CON group in the ventral SGZ region (each group N = 8; for dorsal F_3,28_ = 10.43, p < 0.01; for ventral F_3,28_ = 16.76, p < 0.01; Fig. 5a–c,f).

### Rotarod exercise enhanced the neuronal commitment of the SN and the striatum regions

**Figure 6:**
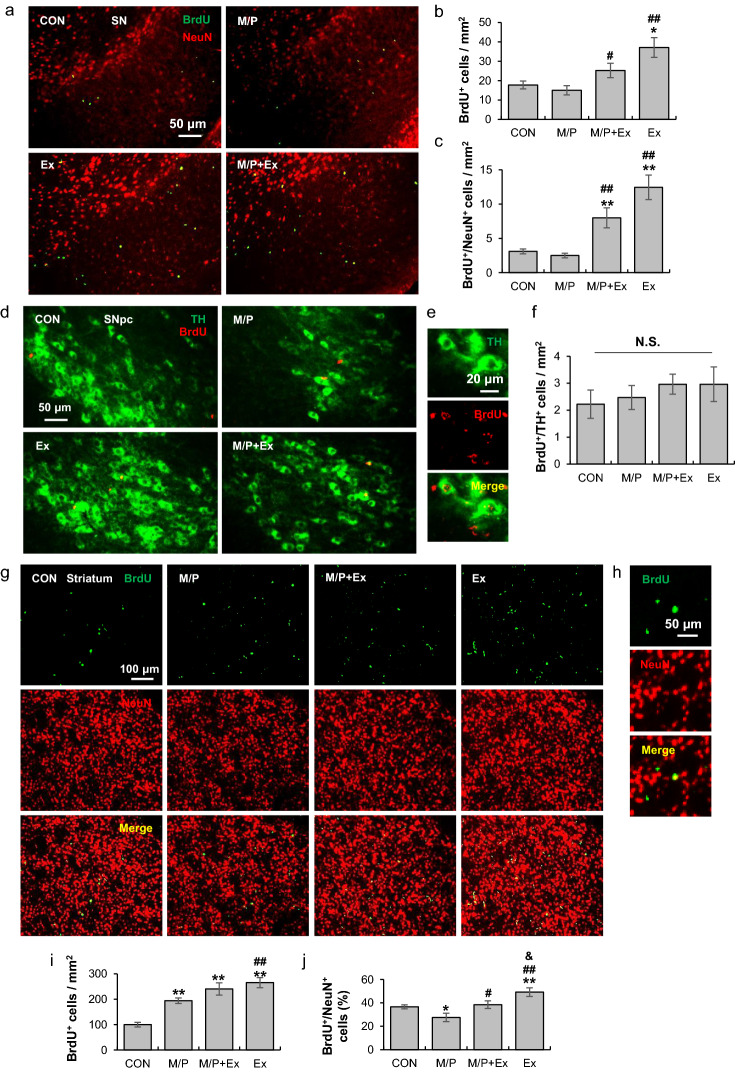
Rotarod walking exercise enhanced the neuronal commitment of the SN and the striatum areas of M/P-treated mice. (**a**) Photomicrographs of the double fluorescent staining of dorsal SN BrdU^+^ and NeuN^+^ cells (CON, N = 8; M/P, N = 9; M/P + Ex, N = 9; Ex, N = 8, 200 × of magnification). (**b**) Quantification of the fluorescent intensity of SN BrdU^+^ cells showing that exercise increased the number of BrdU^+^ cells, regardless of MPTP treatment. (**c**) Quantification of the fluorescent staining of SN BrdU^+^/NeuN^+^ cells showing that exercise increased the number of BrdU^+^/NeuN^+^ cells, regardless of MPTP treatment. (**d**) Photomicrographs of the double fluorescent staining of SNpc BrdU^+^ and TH^+^ cells (N = 9 in each group, 200 × of magnification) showing no difference in the number of BrdU + /TH + cells between groups. (**e**) Representative photomicrograph of the high magnitude of SNpc BrdU^+^/TH^+^ cells (400 × of magnification). (**f**) Quantification of the fluorescent staining of SNpc BrdU^+^/TH^+^ cells. (**g**) Photomicrographs of the double fluorescent staining of striatal BrdU^+^ and NeuN^+^ cells (N = 8 in each group, 200 × of magnification). (**h**) Representative photomicrograph of the high magnitude of striatal BrdU^+^/ NeuN^+^ cells (400 × of magnification). (**i**) Quantification of the fluorescent intensity of striatal BrdU^+^ cells showing that M/P and exercise increased the number of BrdU^+^ cells. (**j**) Quantification of the fluorescent staining of striatal BrdU^+^/NeuN^+^ cells showing that exercise restored the M/P-induced decrease in BrdU^+^/NeuN^+^ cells. Data are presented as mean ± SEM. *p < 0.05, vs. CON; **p < 0.01, vs. CON; ^#^p < 0.05, vs. M/P; ^##^p < 0.01, vs. M/P; ^&^p < 0.05, vs M/P + Ex. SNpc, substantia nigra par compacta.

To evaluate adult neurogenesis in the dopaminergic pathway-related local areas, colocalized BrdU^+^/NeuN^+^ cells were determined in the SN and striatum. In the SN region, exercise enhanced BrdU^+^ and BrdU^+^/NeuN^+^ cells relative to those in CON and M/P mice (CON, N = 8; M/P, N = 9; M/P + Ex, N = 9; Ex, N = 8; for BrdU^+^ F_3,30_ = 7.78, p < 0.01; for BrdU^+^/NeuN^+^ F_3,30_ = 15.53, p < 0.01; Fig. 6a–c). However, BrdU^+^/TH^+^ cells were not different among the four groups (each group N = 9, F_3,32_ = 0.53, p > 0.05, Fig. 6d–f). This result suggests that long-term motor learning training promotes neural precursor cell survival and neuronal commitment in the SN area; however, it does not affect dopaminergic neurogenesis. In the striatum, the number of BrdU^+^ cells in M/P, M/P + Ex, and Ex mice increased significantly compared to those of CON mice. However, M/P decreased the number of BrdU^+^/NeuN^+^ cells, which was restored by exercise. In addition, exercise alone increased BrdU^+^/NeuN^+^ cells (each group N = 8; for BrdU^+^ F_3,28_ = 18.19, p < 0.01; for BrdU^+^ /NeuN^+^ F_3,28_ = 12.73, p < 0.01; Fig. 6g–j).

### Rotarod exercise induced AMPK phosphorylation and BDNF expression in SN area

**Figure 7:**
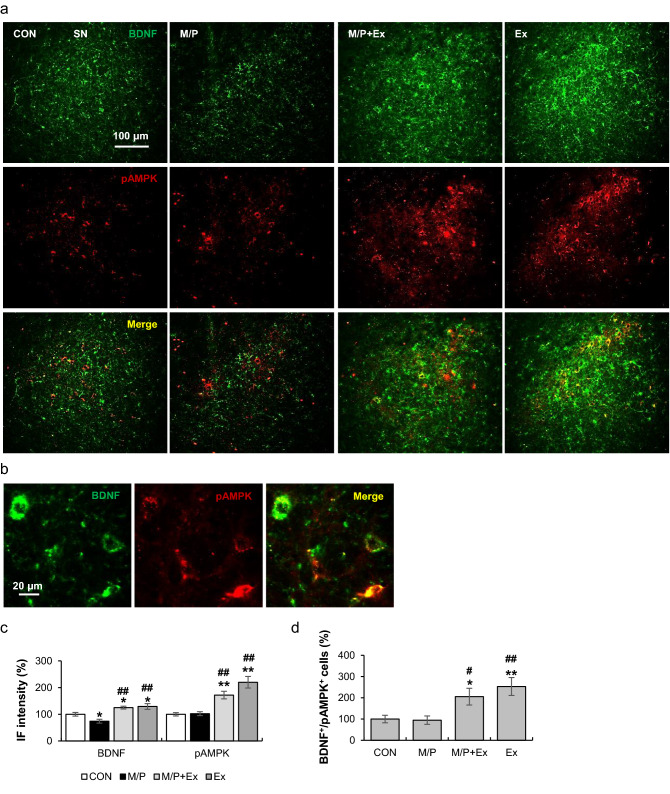
Rotarod walking exercise activated the AMPK/BDNF pathway in the SN area of M/P-treated mice. (**a**) Photomicrographs of the double fluorescent staining of SN BDNF^+^ and pAMPK^+^ cells (N = 8 in each group, 200 × of magnification). (**b**) Representative photomicrograph of the high magnitude of BDNF^+^/pAMPK^+^ cells (400 × of magnification). (**c**) Quantification of the fluorescent intensity of SN BDNF^+^ and pAMPK^+^ cells showing that exercise restored the M/P-induced decrease in BDNF^+^ cells, and enhanced the pAMPK^+^ IF intensity, regardless of M/P treatment. (**d**) Quantification of the fluorescent staining of SN BDNF^+^/pAMPK^+^ cells showing that exercise increased the number of BDNF^+^/pAMPK^+^ cells, regardless of M/P treatment. Data are presented as mean ± SEM. *p < 0.05, vs. CON; **p < 0.01, vs. CON; ^#^p < 0.05, vs. M/P; ^##^ p < 0.01, vs. M/P.

In the SN area, M/P treatment reduced BDNF^+^ immunoreactivity, but not pAMPK^+^ immunoreactivity. However, exercise substantially enhanced the immunoreactivity of BDNF^+^ and pAMPK^+^ regardless of M/P treatment (each group N = 8; for BDNF^+^ F_3,28_ = 10.61, p < 0.1; for pAMPK^+^ F_3,28_ = 17.59, p < 0.01; Fig. 7a–c). Furthermore, BDNF^+^/pAMPK^+^ cells were markedly increased by exercise in the SN area (each group N = 8, F_3,28_ = 6.14, p < 0.01; Fig. 7d).

## Discussion

The present study demonstrated the potential role of motor skill learning training in a subacute MPTP mouse model of PD, particularly by focusing on adult neurogenesis in some areas, including the SVZ, SGZ, SN, and striatum. Four weeks of rotarod walking exercise rescued impaired motor performance and mitigated nigrostriatal dopaminergic degeneration in M/P-treated mice. Regarding adult neurogenesis, exercise intervention exerted pro-neurogenic effects in the M/P-induced perturbation of neurogenesis. In Table 1, we summarize the effects of rotarod exercise and M/P treatment on the proliferation, differentiation, and survival of neural precursor cells using specific markers, such as Ki67, DCX, and BrdU. Notably, M/P treatment affected adult neurogenesis in a region-specific manner, while the long-term rotarod exercise exerted a pro-neurogenic effect in two chief neurogenic niches (SVZ and SGZ) and local regions, including the striatum and SN. Furthermore, the exercise regimen facilitated AMPK activation and BDNF expression in the regions identical/adjacent to neurogenic areas assessed in this study.

**Table 1:** Summary of the proliferation, neuronal differentiation, and survival capacity of neural precursor cells in response to M/P and/or rotarod walking exercise.

Proliferation	Group			
Ki67^+^	CON	M/P	M/P + Ex	Ex
**Area**				
SVZ	–	**↑**	**↑**	**↑**
dSGZ	–	–	**↑**	**↑**
vSGZ	–	–	**↑**	**↑**

Neuronal differentiation	Group			
DCX^+^ or BrdU^+^/NeuN^+^	CON	M/P	M/P + Ex	Ex
**Area**				
SVZ	–	**↓**	–	–
dSGZ	–	–	**↑**	**↑**
vSGZ	–	**↓**	**↑**	**↑**
SN	–	–	**↑↑**	**↑↑**
Striatum	–	**↓**	–	–

Progenitor cell survival	Group			
BrdU^+^	CON	M/P	M/P + Ex	Ex
**Area**				
SVZ	–	**↑**	**↑**	**↑**
dSGZ	–	–	**↑**	**↑**
vSGZ	–	–	**↑↑**	**↑↑**
SN	–	–	–	**↑**
Striatum	–	**↑**	**↑↑**	**↑↑**

(–: basal levels, ↑: 1.5–twofold increase, ↑↑: increase more than twofold, ↓: 1.5 fold decrease).

In clinical practice, exercise and locomotor training are used as physical interventions for neurorehabilitation in PD, and primarily target goal-directed motor skill learning to improve gait performance and dynamic balance. For example, a 6-week of intensive treadmill training or an 8-week of perturbation treadmill walking with small three-dimensional tilting movements of the walking surface improved gait rhythmicity and postural stability in patients^31,32^. Moreover, an exercise-based adapted Tango program improved the clinical measures of gait and balance by improving muscle coordination (i.e., more sensitive motor module distinctness and consistency) associated with motor skill reacquisition in subjects with PD^33^. The results of these previous studies provide evidence for the movement-restoring role of motor skill learning training in PD-associated impaired motor performance. In connection with motor skill learning training in rodents, the rotarod test is commonly used to measure motor performance, including motor coordination and learning in cerebellar-defective or PD rodent models^34–37^. Rotarod training also resulted in structural plasticity in various brain regions, as measured by multimodal MRI^38^. Furthermore, basal ganglia circuity is a crucial neuro-network in serial procedures of motor skill learning^39^, and nigrostriatal dopaminergic defects cause deficient motor skill learning^40^. Based on this rationale, we adopted a 4-week rotarod walking exercise with a weekly progressive increment of rotation speed to increase task complexity as a motor skill learning training. In this study, rotarod walking exercise effectively reduced MPTP-induced impairment of motor coordination, dyskinesia, and reduction of muscular endurance. The behavioral data was in line with the results of the afore-mentioned studies, suggesting that our exercise regimen is an appropriate motor skill learning training in a PD mouse model.

The behavioral data obtained from this study was in line with the histopathological changes, in which the exercise regimen improved nigrostriatal dopaminergic degeneration in MPTP-treated mice. Furthermore, activated AMPK and upregulated BDNF expression was verified in the SN area in our experimental paradigm. Previous studies suggest that the neuroprotective effects of physical exercise as a neurorehabilitation strategy in PD are associated with upregulated neurotrophic factors, such as BDNF and GDNF^23,24^. Moreover, AMPK activation contributes to lowering α-synuclein phosphorylation and inducing BDNF expression in subchronic MPTP-treated mice^41,42^. A recent study showed that activating the AMPK/CREB pathway facilitated TH-activated DA generation and upregulated neurotrophic factors, including BDNF, in metformin-administered mice and SH-SY5Y cells^43^. Furthermore, emerging evidence from animal studies demonstrated that the exercise-elicited AMPK/BDNF pathway plays a crucial role in modifying diseases, such as depression, memory deficiency, and AD^44–46^. Therefore, our data, along with previous findings, suggest that activation of the nigral AMPK/BDNF pathway induced by rotarod walking exercise may be helpful in protecting DAergic cells and their innervation, thereby having a beneficial impact on the cortico-basal ganglia circuit responsible for automatic motor control.

Along with the basal ganglia governing cardinal motor symptoms, PD pathophysiology has been reported to affect broad neuronal structures^6^. Disrupted adult neurogenesis in two main neurogenic niches (SVZ and SGZ) is implicated in olfactory dysfunction, cognitive decline, and psychiatric abnormalities prior to the manifestation of motor symptoms in PD progression^4,10,11,13^. Animal studies have shown conflicting results, indicated by increased, decreased, or unchanged adult neurogenesis in MPTP-injected animals^10,11,47^. This discrepancy may be attributed to the modality of MPTP injection (acute, subchronic, or chronic) and the evaluation time after MPTP injection. In the present study, we found that the proliferative capacity of neural precursor cells was region-specific in the SVZ (increased) and the SGZ (unchanged), while the differentiation into neurons in both areas was attenuated in the context of MPTP-toxicity. Particularly, in the SGZ, neuronal differentiation in the ventral part was more vulnerable to MPTP than the dorsal part. Since the SVZ and the DG receive DAergic innervation derived from the SN^10,48,49^, reduced neurogenesis in both regions is thought to be attributed to DAergic nigral deafferentation. In addition, the MPTP-induced increase in proliferative capacity in the SVZ may be a putative compensatory mechanism for the enhanced self-repairing process in cell death^50,51^. Therefore, the perturbation of neurogenesis via the deficits of DA neurotransmission into both the SVZ and DG may be implicated in non-motor symptoms, including hyposmia and psychiatric disturbance in PD progression^4,6,13^. In contrast, exercise regimens exert pro-neurogenic effects in both the SVZ and SGZ, and AMPK appears to play a crucial role in promoting adult neurogenesis^52,53^. Several lines of evidence have described the pro-neurogenic role of BDNF in the SGZ and SVZ; however, there is a region-specific difference in its cognate receptor system, including BDNF/Trk in the SGZ or BDNF/p75NTR in the SVZ^54–56^. Based on the previous studies, our findings suggest that nigral AMPK-activated BDNF upregulation may be an indispensable mechanism underlying the neuroplastic role of motor skill learning training in PD-linked aberrant neurogenesis in the SVZ and SGZ regions.

In addition to the two major neurogenic niches, a few studies have reported the existence of SN adult neurogenesis via the migration of DAergic neurons derived from stem cells lining the cerebroventricular system in the midbrain or the occurrence of DAergic neurogenesis in the SN area per se^17,18,57^, speculating that newly generated neurons could be replenished in the SN area within the life span of the mouse. Based on these data, we hypothesized that the inequivalent rate between nigral neurogenesis (decline) and cell death (increment) may contribute to progressive DAergic cell loss in PD. A previous study reported that MPTP-exposed partial insults increased the number of BrdU^+^/TH^+^ cells^18^. However, we observed no difference in the number of BrdU-labeled cells expressing TH in MPTP-treated SN, which was in line with the result of another study^19^. The MPTP-induced increase in BrdU^+^/TH^+^ cells was only observed with high BrdU incorporation, since the low number of newborn cells in the adult SN^18^, which may explain the discrepancy between our results and others’ results according to the difference in BrdU dosing (Zhoa lab.: the consumption using drinking water containing 100 mg/kg, 1 mg/mL of BrdU for 2–6 weeks or the continuous infusion of 50–150 mg/mL of BrdU using osmotic pump for 21 days vs. ours: the intraperitoneal injection of 50 mg/kg/day for 5 days). Nevertheless, our exercise regimen resulted in a robust increase in nigral BrdU^+^/NeuN^+^ cells, along with augmented AMPK and BDNF. A previous study revealed that an enriched environment with physical activity caused an apparent increase in neurogenesis and gliogenesis in the adult SN^58^. Previous studies, as well as our results, partly support our hypothesis that motor skill learning training may affect AMPK/BDNF-modulated facilitation of nigral adult neurogenesis.

The survival capacity of neural precursor cells is increased by MPTP exposure in the striatum. This change may be a compensatory effect to overcome the noxious environment such as DAergic denervation. However, our exercise intervention improved the MPTP-induced decrease in striatal BrdU-labeled neurons (BrdU^+^/NeuN^+^ cells; neuronal differentiation). Although the origin of adult striatal neurogenesis is controversial, most evidence suggests migration from the SVZ^59,60^. Furthermore, DA is an important regulator of adult neurogenesis in PD, and neural precursor cells have been reported to express DA receptors in the SVZ^61,62^. The striatal neurogenic change correlated with that of the SVZ area in our study. Furthermore, exercise intervention restored MPTP-induced striatal dopaminergic denervation and enhanced SVZ neurogenic capacity via the AMPK/BDNF pathway. In this study, the expressions of neurogenic-related markers, AMPK, and BDNF proteins were mostly assessed using histological immunostaining to analyze their region-specific expressions. However, additional experimental techniques, including biochemical analysis, such as western blotting, and gene manipulation technique, such as knockdown or overexpression, may be necessary to allow further validation of our findings. Taken together, the exercise-modified striatal neurogenic alternation in MPTP toxicity may be due to striatal DA re-innervation and enhanced SVZ neurogenesis.

In conclusion, our data support the hypothesis that motor skill learning training in PD-linked nigrostriatal degeneration and aberrant neurogenesis in the affected regions is associated with neuroprotective and neuroplasticity-promoting effects. Therefore, motor skill learning training may be useful in improving motor symptoms and improving PD pathophysiology by stimulating the cellular microenvironment and promoting neuroplasticity.

## Data Availability

The datasets used and/or analyzed during the current study are available from the corresponding author on reasonable request.

## Abbreviations

AD: Alzheimer’s disease
AMPK: Adenosine monophosphate-activated protein kinase
BDNF: Brain-derived neurotrophic factor
BrdU: 5-Bromo-2′-deoxyuridine
CREB: CAMP response element binding protein
DA: Dopamine
DCX: Doublecortin
DG: Dentate gyrus
GDNF: Glial cell-derived neurotrophic factor
MPTP: 1-Methyl-4-phenyl-1,2,3,6-tetrahydropyridine
PaGE: Paw grip endurance test
PD: Parkinson’s disease
SGZ: Subgranular zone
SN: Substantia nigra
SVZ: Subventricular zone
TH: Tyrosine hydroxylase
